# Molecular Hydrogen as a Regulator of Mitochondrial Quality Control and Metabolic Reprogramming

**DOI:** 10.3390/ijms27114948

**Published:** 2026-05-29

**Authors:** Fangfang Li, Yue Jing, Chao Xia, Ruping Zhao, Mengyu Liu, Pengxiang Zhao, Xuemei Ma, Fei Xie

**Affiliations:** College of Chemistry and Life Sciences, Beijing University of Technology, Beijing 100124, China; 16638421128@163.com (F.L.); jingyue0525@163.com (Y.J.); xiac2025@163.com (C.X.); 15732855958@163.com (R.Z.); mengyu@bjut.edu.cn (M.L.); zpx@bjut.edu.cn (P.Z.)

**Keywords:** molecular hydrogen, mitochondrial quality control, redox homeostasis

## Abstract

The biological effects of molecular hydrogen are moving beyond the traditional explanatory framework of “selective antioxidation.” This article systematically integrates the basic, preclinical, and preliminary clinical evidence for hydrogen in metabolic diseases, neurodegenerative disorders, and cancer, centering on the two major themes of mitochondrial quality control and metabolic reprogramming. Current studies indicate that hydrogen can reshape redox homeostasis, coordinate mitochondrial biogenesis, dynamic balance, and mitophagy, and modulate key signaling axes such as AMPK/Sirtuins, PGC-1α, and PPARα, aimed at optimizing mitochondrial function, thereby influencing adaptive glucose and lipid metabolism as well as cellular bioenergetic homeostasis. Although its upstream initiating events and context dependency remain to be clarified, existing evidence supports the view that hydrogen is an important network regulator linking redox regulation, mitochondrial homeostasis, and metabolic adaptation.

## 1. Introduction

Since Ohsawa et al. reported in 2007 that H_2_ exerts antioxidant and tissue-protective effects, H_2_ has attracted widespread attention as a potential medical gas in a variety of disease models [1,2,3]. Early studies largely explained its effects in terms of “selective antioxidation,” proposing that H_2_ preferentially attenuates oxidative damage associated with hydroxyl radicals and peroxynitrite while minimally interfering with physiological redox signaling [1,2]. However, accumulating evidence suggests that simple free radical scavenging is insufficient to fully account for its broad-spectrum and relatively sustained biological effects [2,3]. It is now generally believed that H_2_ may exert systemic protective effects by modulating reactive oxygen species (ROS)/reactive nitrogen species (RNS), lipid peroxidation chain reactions, and their derived signaling mediators, thereby further influencing inflammatory responses, transcriptional regulation, and metabolic networks [2,4].

A central theoretical challenge currently facing this field is the “stoichiometric mismatch.” The steady-state concentration of exogenously administered hydrogen in vivo is usually only at the micromolar (μM) level, and because of its extremely high diffusibility, its residence time in the body is very short [5,6]. In contrast, the total amount of ROS generated under pathological conditions is enormous. From a stoichiometric perspective, neither the second-order rate constant for the continuous reaction of hydrogen with free radicals nor its instantaneous concentration is sufficient to explain its broad and sustained regulatory effects at the transcriptomic, proteomic, and metabolomic levels [7,8].

At the same time, a growing body of research indicates that the biological effects of long-term or low-dose hydrogen intervention cannot be fully explained by “direct free radical scavenging” alone. Beyond its antioxidant effects, hydrogen may also influence NAD(P)H-related redox status, mitochondrial function, and stress-response pathways such as Nrf2, thereby reshaping cellular redox and metabolic networks at a higher level [2,3,4]. Notably, these effects do not necessarily depend on direct chemical reactions between hydrogen and target molecules, but are more likely to involve indirect remodeling of free radical chain reactions, changes in lipid peroxidation-derived mediators, and mitochondria-related signaling networks [2,4]. Therefore, hydrogen may possess some characteristics of a gaseous signaling regulator and may indirectly participate in the regulation of metabolic adaptation by influencing mitochondrial homeostasis and redox signal transduction. However, based on the current evidence, it is still insufficient to define hydrogen as a confirmed “gasotransmitter” or “protein allosteric regulator” [2,3].

Based on the above understanding, the academic community’s interpretation of the mechanisms of hydrogen is gradually expanding from a framework of “passive free radical scavenging” to a more integrated paradigm of “active regulation of the redox–mitochondria–metabolism network.” However, at the present stage, it is more appropriate to regard metabolic reprogramming as one of the major mechanistic themes of hydrogen, rather than as its sole definitively established core mechanism [3,9]. Accordingly, this article focuses on how hydrogen may be linked to MQC, energy sensing, and metabolic adaptation, with particular emphasis on the potential roles of the AMPK/Sirtuins axis, mitochondrial biogenesis, dynamic balance, and mitophagy, as well as their relationships with disordered glucose and lipid metabolism under pathological conditions. It should be emphasized that there is still no consistent and direct evidence demonstrating whether hydrogen can directly activate AMPK, induce the mitochondrial unfolded protein response (UPRmt), or stably drive a metabolic shift from pathological glycolysis toward oxidative phosphorylation (OXPHOS) [2,3,10]. This mitochondria- and metabolism-centered research framework provides a more systematic theoretical basis for understanding the potential applications of hydrogen in metabolic diseases, neurodegenerative disorders, and cancer, although its upstream initiating events and context dependency remain to be further elucidated [2,3,9].

## 2. Routes of Hydrogen Administration and Tissue Pharmacokinetic Characteristics

Before discussing the molecular mechanisms by which hydrogen regulates mitochondrial function and metabolic reprogramming, it is necessary to first clarify the pharmacokinetic characteristics, tissue distribution patterns, and applicable scenarios associated with different routes of administration. Existing studies indicate that the biological effects of H_2_ are influenced by factors such as the route of administration, exposure duration, local tissue concentration, and model type, which may partly explain the heterogeneity among different study findings. For example, in LPS-induced acute lung injury or inflammatory injury models, hydrogen inhalation, oral HRW, and hydrogen-rich saline injection have all been used to evaluate the protective effects of hydrogen; however, the intervention forms, doses, exposure durations, and outcome measures vary across studies [11,12,13,14]. Therefore, when comparing findings from different studies or interpreting the mitochondrial protective mechanisms of hydrogen, it‘s in vivo exposure characteristics should be distinguished according to the route of administration.

At present, the main approaches for hydrogen intervention include hydrogen inhalation, oral HRW, and hydrogen-rich saline injection. Hydrogen inhalation is one of the most direct approaches for achieving rapid systemic exposure. Its major advantage is that hydrogen can rapidly enter the bloodstream through alveolar gas exchange and subsequently be distributed to tissues throughout the body via the circulation. Pharmacokinetic studies in porcine models have shown that inhaled hydrogen can rapidly enter the arterial circulation; after cessation of inhalation, blood hydrogen concentrations decline rapidly, suggesting a dynamic pattern of “rapid exposure–rapid clearance” [15]. Real-time monitoring of tissue hydrogen concentrations further indicates that, after continuous hydrogen inhalation, the rate of increase and peak concentration of hydrogen differ among organs and tissues, suggesting an organ-dependent pattern of tissue distribution [16]. Therefore, hydrogen inhalation is more suitable for studies requiring rapid systemic exposure, such as acute inflammatory injury, ischemia–reperfusion injury, or critical illness interventions. However, its application depends on specialized gas delivery equipment, and inhaled concentration, exposure duration, safety, and dose control must be strictly standardized [15,16,17,18].

Compared with hydrogen inhalation, oral HRW is more suitable as a noninvasive and repeatable long-term intervention. Because hydrogen has limited solubility in water and readily escapes during storage and after opening, the actual exposure level of HRW is affected by preparation methods, storage conditions, drinking volume, and intake frequency. Studies measuring breath hydrogen in humans have shown that, after ingestion of HRW, breath hydrogen levels rise within a short period, peak at approximately 10–15 min, and then decline rapidly, suggesting that oral HRW produces a relatively transient, pulse-like exposure pattern [19]. Animal pharmacokinetic studies have also shown that, after gastrointestinal administration of hydrogen-rich solution, portal venous hydrogen concentrations may increase, whereas peripheral arterial hydrogen levels do not necessarily rise in parallel, suggesting that HRW may predominantly produce local exposure along the gastrointestinal–portal vein–liver axis [20]. Therefore, HRW may be more suitable for studies of chronic metabolic disorders, inflammation-related diseases, or long-term preventive interventions, but it is not appropriate as an emergency administration approach requiring rapid and high-level systemic exposure [17,18,19,20,21,22].

Hydrogen-rich saline injection is another commonly used administration method in experimental studies. It can partially bypass gastrointestinal absorption and improve the operability of dose control. Depending on the specific experimental purpose, hydrogen-rich saline can be administered via intraperitoneal injection, intravenous injection, or local delivery. Pharmacokinetic studies of intraperitoneal injection have shown that hydrogen-rich saline can increase hydrogen concentrations in the inferior vena cava, whereas hydrogen concentrations in the carotid artery increase only slightly, suggesting route-dependent exposure and local distribution after intraperitoneal administration [23]. Studies on intravenous infusion of hydrogen-rich saline further suggest that hydrogen-rich liquid formulations can be used to establish a relatively controllable blood exposure model, although their clinical translation still requires further pharmacokinetic, safety, and efficacy evidence [24]. In terms of application scenarios, hydrogen-rich saline is mainly used in preclinical organ injury models, such as endotoxin-induced lung injury, intestinal ischemia–reperfusion injury, renal ischemia–reperfusion injury, and cerebral ischemic injury [13,23,25,26]. Therefore, the advantage of this administration route lies in its relatively controllable dosing under experimental conditions; however, it still has considerable limitations, mainly including its invasive nature, the need for sterile preparation, and insufficient clinical feasibility and standardization.

In summary, different hydrogen administration routes have distinct pharmacokinetic characteristics and tissue exposure patterns. Hydrogen inhalation offers the advantage of rapid systemic exposure and is suitable for acute injury or short-term interventions. Oral HRW is noninvasive and repeatable over the long term, making it more suitable for chronic diseases and metabolic regulation studies. Hydrogen-rich saline injection is more commonly used in preclinical organ injury models and experimental studies requiring stricter dose control. Given the substantial differences among current studies in hydrogen concentration, exposure duration, tissue detection methods, and endpoint indicators, it is not yet appropriate to define a unified “minimum effective concentration” or “effective threshold.” Future studies should integrate real-time monitoring of tissue hydrogen concentrations, standardized dosing regimens, and mitochondrial functional endpoints to establish a clearer dose–exposure–response relationship, thereby providing a basis for the translation of hydrogen therapy from basic research to clinical application [15,16,17,18,20,23,24].

As summarized in Table 1, the three commonly used routes of H_2_ administration—hydrogen inhalation, oral HRW, and hydrogen-rich saline injection—show distinct pharmacokinetic characteristics, tissue distribution patterns, application scenarios, and limitations. Hydrogen inhalation enables rapid pulmonary absorption and systemic exposure, making it suitable for acute inflammatory injury, ischemia–reperfusion injury, and critical illness models that require rapid delivery [15,16,17,18]. Oral HRW provides a noninvasive and repeatable intervention strategy, but its exposure level is affected by dissolved H_2_ concentration, preparation method, storage conditions, intake volume, and intake frequency [17,18,19,20,21,22]. Hydrogen-rich saline injection allows relatively controlled dosing in experimental settings and is widely used in preclinical organ injury models, although its invasive nature, requirement for sterile preparation, and limited clinical standardization should be considered [13,23,24,25,26].

## 3. Mitochondrial Quality Control (MQC): Cellular Energy Conversion

### 3.1. Hypothesis of Hydrogen Transmembrane Diffusion and Quantum Regulation of the Electron Transport Chain

H_2_ is the smallest naturally occurring nonpolar diatomic molecule. Its unique physicochemical properties allow it, in accordance with Fick’s law, to freely penetrate multiple lipid bilayer barriers, including the inner mitochondrial membrane (IMM), with an exceptionally high diffusion coefficient. In this way, it can overcome the steric hindrance and transport limitations faced by conventional macromolecular antioxidants and directly reach the core functional region of the electron transport chain (ETC) deep within the mitochondrial matrix [15]. Although direct experimental evidence for mitochondrial uptake of hydrogen remains inconclusive, existing studies have clearly shown that hydrogen can mediate a series of key biological effects and signaling pathways through functional regulation of mitochondria, thereby exerting profound effects on cellular metabolism. Based on the evolutionary homology between mitochondrial respiratory chain Complex I (NADH oxidoreductase) and [NiFe]-hydrogenase, Ishibashi et al. proposed that H_2_ may directly act on the ubiquinone-binding chamber of Complex I. Under pathological conditions, by intervening in electron and proton transfer, hydrogen may “rectify” disordered electron flow and thereby suppress superoxide generation at its source. This mechanism provides a deeper molecular basis for understanding the biological effects of hydrogen in regulating mitochondrial function [30]. In a 2020 study, Ishihara et al. [31] further demonstrated through high-resolution enzyme kinetic analysis that hydrogen can specifically inhibit superoxide anion leakage mediated by iron–sulfur (Fe–S) clusters in Complex I, while exerting no inhibitory effect on normal OXPHOS-associated electron transport, thereby preserving the mitochondrial membrane potential (ΔΨm).

Given that electron transfer between the iron–sulfur clusters in Complex I is widely considered to depend on long-range quantum tunneling, together with evidence that hydrogen may serve as an electron/proton donor for this complex, rectify electron flow, and suppress ROS generation [30,31,32], some scholars have speculated that hydrogen may fine-tune the barrier and pathway of proton-coupled electron transfer (PCET) by altering the microenvironment surrounding the cofactors. Although there is currently no direct experimental evidence demonstrating that hydrogen can specifically regulate the electronic spin states of iron–sulfur clusters or the local dielectric constant, this “electronic rectification–quantum fine-tuning” hypothesis offers a possible quantum-biological explanation for how hydrogen at micromolar concentrations may precisely interfere with upstream ROS generation in the ETC [31].

**Figure 1:**
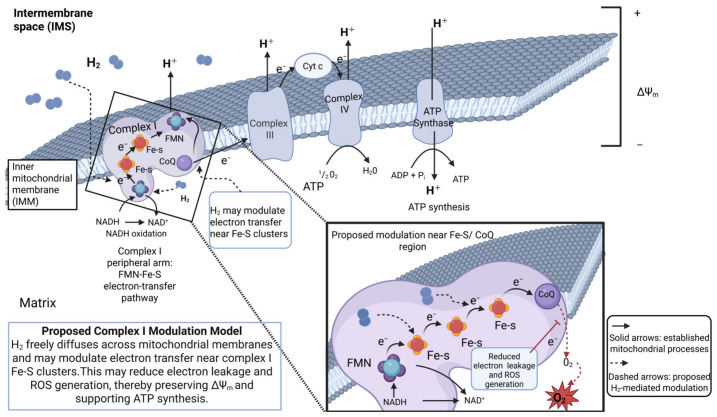
Proposed model of H_2_-mediated modulation of electron transfer at mitochondrial respiratory chain complex I.

As shown in Figure 1, mitochondrial respiratory chain complex I is a key entry point for NADH-derived electrons. NADH is oxidized to NAD^+^ at complex I, and electrons are transferred through the FMN–Fe–S pathway toward CoQ. Based on the proposed model, H_2_ may diffuse across mitochondrial membranes and modulate electron transfer near the Fe–S/CoQ region of complex I, thereby reducing electron leakage and ROS generation. This process may contribute to the preservation of mitochondrial membrane potential (ΔΨm) and ATP synthesis.

H_2_ can freely diffuse across mitochondrial membranes and may modulate electron transfer near the Fe–S/CoQ region of mitochondrial respiratory chain complex I. In the established respiratory process, NADH is oxidized to NAD^+^ at complex I, and electrons are transferred through the FMN–Fe–S pathway to CoQ and subsequently to downstream respiratory complexes. Complexes I, III, and IV contribute to proton pumping across the inner mitochondrial membrane, whereas ATP synthase uses the proton gradient to support ATP synthesis. The proposed H_2_-mediated modulation may reduce electron leakage and ROS generation, thereby helping preserve mitochondrial membrane potential (ΔΨm) and mitochondrial bioenergetics. Solid arrows indicate established mitochondrial processes, whereas dashed arrows indicate proposed H_2_-mediated modulation.

### 3.2. Redox-Driven Optimization of Mitochondrial Bioenergetic Metabolism

Mitochondrial respiratory chain function is central to the maintenance of cellular energy homeostasis, and the optimizing effect of hydrogen on mitochondrial bioenergetic metabolism is first manifested in its direct or indirect regulation of ETC complex activity. Multiple studies have shown that under pathological conditions such as ischemia–reperfusion and sepsis, hydrogen intervention can improve mitochondrial ETC function and, in some models, restore the suppressed activities of Complex I and Complex II, accompanied by an increase in ΔΨm and ATP production, thereby alleviating mitochondrial dysfunction [33]. The neuroprotective effects of H_2_ depend on specific biological signaling pathways rather than on mere physical effects. Studies have shown that its antioxidant efficacy in models of subarachnoid hemorrhage (SAH) strictly depends on activation of the Nrf2 signaling axis and is mediated through this pathway by the induction of programmed mitophagy [34]. As a central regulator of the cellular antioxidant stress response, activation of Nrf2 can upregulate the expression of heme oxygenase-1 (HO-1) and mitochondrial superoxide dismutase 2 (SOD2), thereby maintaining redox balance within the mitochondrial microenvironment.

To establish the causal relationship within the “ROS–Nrf2–mitochondria” axis, many research groups have conducted validation experiments using Nrf2 knockout (Nrf2^−/−^) mouse models [37,38,39,40]. The results showed that hydrogen effectively reversed sepsis-induced suppression of state 3 mitochondrial respiration in wild-type mice; however, in Nrf2^−/−^ mice, this mitochondrial protective effect of hydrogen was largely abolished, and hydrogen failed to significantly ameliorate mitochondrial swelling or collapse of the ΔΨm [33]. This key evidence indicates that the optimization of mitochondrial bioenergetic metabolism by hydrogen has a clear molecular dependency—based on the models studied to date, this effect depends to a large extent on activation of the Nrf2 pathway, through which Nrf2 preserves the structural integrity and catalytic activity of respiratory chain enzyme complexes by enhancing mitochondrial antioxidant capacity and quality control.

At present, clinical evidence regarding the effects of H_2_ on mitochondrial function remains relatively limited, and a systematic and well-established body of research has yet to be formed. In the future, with continuous optimization of clinical study design and expansion of sample sizes, it is expected that the biological effects and clinical value of H_2_ can be further explored in greater depth from the perspectives of mitochondrial regulatory mechanisms and functional restoration, thereby providing a more solid clinical basis for its rational application in the prevention and treatment of related diseases.

### 3.3. Mitochondrial Biogenesis and Dynamics Remodeling

Mitochondrial biogenesis is a key process for maintaining mitochondrial abundance, structural integrity, and metabolic adaptability, and one of its core regulators is peroxisome proliferator-activated PGC-1α [33,36]. PGC-1α primarily functions as a transcriptional coactivator and promotes mtDNA replication, mitochondrial protein expression, and mitochondrial biogenesis by regulating downstream molecules such as NRF1/2 and mitochondrial transcription factor A (TFAM) [33]. It should be noted that NRF1/2 here refers to the nuclear respiratory factors involved in mitochondrial biogenesis, which are distinct from Nrf2/NFE2L2 in the antioxidant stress response pathway [33].

Existing studies indicate that H_2_ may promote adaptive mitochondrial repair under pathological conditions through PGC-1α-related pathways [33,36]. In a sepsis-associated brain injury model, H_2_ inhalation improved neurological and mitochondrial function and upregulated PGC-1α and its downstream mitochondrial biogenesis-related factors, suggesting that PGC-1α may serve as an important regulatory node in H_2_-mediated mitochondrial functional recovery [33]. In a myocardial ischemia–reperfusion injury model, H_2_ treatment similarly increased PGC-1α expression and improved myocardial mitochondrial structure and function; moreover, PGC-1α deficiency weakened the protective effect of H_2_ against ischemia–reperfusion-induced myocardial injury, further supporting the functional significance of PGC-1α in H_2_-mediated mitochondrial protection [36]. Therefore, under specific injury or metabolic stress conditions, H_2_ may participate in mitochondrial functional recovery and adaptive energy metabolism by activating the PGC-1α–NRF1/2–TFAM-related mitochondrial biogenesis program [33,36].

In addition to mitochondrial biogenesis, mitochondrial dynamics remodeling is also an important component through which H_2_ maintains mitochondrial homeostasis [25,26,35,36]. Mitochondrial fission and fusion jointly determine mitochondrial network morphology, maintenance of membrane potential, respiratory function, and ATP-generating capacity [35,36]. Under pathological conditions, excessive fission and mitochondrial fragmentation are usually accompanied by increased oxidative stress, decreased membrane potential, and impaired energy metabolism [35,36]. Recent studies have shown that high-concentration H_2_ inhalation can improve mitochondrial dynamics abnormalities and alleviate neurological injury in mice with sepsis-associated encephalopathy [35]. In myocardial ischemia–reperfusion-related studies, H_2_ has also been shown to regulate mitochondrial dynamics-related proteins, including dynamin-related protein 1 (Drp1), mitochondrial fission factor (MFF), mitofusin 1/2 (Mfn1/2), and optic atrophy 1 (OPA1), thereby improving mitochondrial structural damage and cellular stress responses [36].

From a mechanistic perspective, the effects of H_2_ on mitochondrial biogenesis and mitochondrial dynamics may not be independent of each other [33,35,36]. PGC-1α-related mitochondrial biogenesis can enhance mitochondrial functional reserve, whereas regulation of fission–fusion proteins such as Drp1 and Mfn2 helps maintain the integrity and functional continuity of the mitochondrial network [33,35,36]. These processes may jointly participate in MQC under pathological stress conditions; however, the upstream direct targets through which H_2_ regulates PGC-1α, Drp1, and Mfn2 remain incompletely defined, and related findings may be influenced by tissue type, disease model, route of H_2_ administration, and intervention duration [33,35,36].

Overall, the improvement of mitochondrial homeostasis by H_2_ may involve two interrelated aspects: on the one hand, promoting mitochondrial biogenesis through the PGC-1α–NRF1/2–TFAM-related pathway; and on the other hand, regulating the Drp1/Mfn2-related fission–fusion balance in specific injury models, thereby improving mitochondrial structural integrity and metabolic adaptability [33,35,36]. Future studies should integrate tissue-specific expression profiling, mtDNA copy number assessment, high-resolution mitochondrial respirometry, quantitative analysis of mitochondrial morphology, and genetic intervention models to further clarify the direct molecular targets and disease specificity of H_2_ in regulating mitochondrial biogenesis and dynamics remodeling [33,35,36].

### 3.4. Mitophagy and Clearance of Damaged Mitochondria

Mitophagy is an important component of MQC, which selectively identifies, sequesters, and degrades dysfunctional or irreversibly damaged mitochondria, thereby preventing damaged mitochondria from continuously producing excessive ROS and releasing pro-apoptotic signals [27,41]. Mitophagy is not an isolated terminal event of MQC; rather, together with mitochondrial biogenesis, mitochondrial dynamics remodeling, proteostasis, and the lysosomal degradation system, it constitutes a core process for the dynamic renewal of the mitochondrial pool and plays an important role in maintaining energy metabolism, cellular homeostasis, and aging- or disease-related mitochondrial adaptive responses [27,42].

Current studies suggest that, under oxidative stress conditions such as ischemia–reperfusion-like injury, inflammatory injury, and sepsis-associated organ injury, H_2_ may participate in mitochondrial protection by regulating PTEN-induced kinase 1 (PINK1)/Parkin-related mitophagy [28,43]. The canonical PINK1/Parkin pathway is usually triggered by mitochondrial damage or loss of mitochondrial membrane potential; PINK1 becomes stabilized on the outer membrane of damaged mitochondria, subsequently recruits the E3 ubiquitin ligase Parkin, promotes ubiquitination of outer mitochondrial membrane proteins, and mediates the clearance of damaged mitochondria through autophagy receptors, LC3-related mechanisms, and the lysosomal system [27,42]. Accordingly, under specific pathological conditions, H_2_ may alleviate mitochondrial dysfunction and oxidative stress injury by restoring or enhancing the clearance capacity for damaged mitochondria [28,43].

In a hippocampal neuronal model of oxygen-glucose deprivation/reoxygenation (OGD/R) injury, H_2_ improved cell viability, reduced oxidative stress and mitochondrial functional damage, and upregulated mitophagy-related markers, including PINK1, Parkin, and LC3 [28]. These findings suggest that PINK1/Parkin-related mitophagy may participate in the neuroprotective effects of H_2_, although further assessment of mitophagic flux is still required to confirm this dynamic process [28,41]. In a model of sepsis-associated acute lung injury, Chen et al. provided mechanistic evidence from both in vitro and in vivo experiments, showing that H_2_ ameliorated LPS-induced injury in RAW 264.7 macrophages and enhanced mitophagy [43]. PINK1 knockdown attenuated the protective effects of H_2_ on mitophagy and cellular injury, suggesting that PINK1-mediated mitophagy plays an important role in the protective effects of H_2_ [43]. In mice subjected to cecal ligation and puncture (CLP), H_2_ also alleviated acute lung injury by activating PINK1-mediated mitophagy [44]. Together, these findings support PINK1/Parkin-related mitophagy as a possible mechanism underlying H_2_-mediated MQC, although its applicability should still be interpreted in the context of disease type, tissue background, and injury severity [28,42,43].

From a methodological perspective, transmission electron microscopy (TEM) can directly visualize the ultrastructural morphology of mitochondria enclosed by double-membrane autophagic structures and is therefore an important method for assessing morphological evidence of mitophagy [41]. However, an increase in the number of autophagosomes or mitophagosomes observed by TEM alone cannot independently prove enhanced mitophagic flux [41]. Evaluation of mitophagic flux should be supported by multidimensional evidence, including PINK1/Parkin translocation, LC3-II conversion, p62/SQSTM1 degradation, mitochondria–lysosome colocalization, lysosomal inhibitor treatment, or fluorescence reporter systems [41]. Therefore, in studies that provide only morphological evidence, even if damaged mitochondria are observed to be enclosed by autophagic structures, this can only suggest possible activation of mitophagy and cannot directly demonstrate increased mitophagic flux [41].

The regulation of mitophagy by H_2_ is markedly context-dependent [28,42,43]. Moderate enhancement of PINK1/Parkin-related mitophagy may help remove irreversibly damaged mitochondria, reduce ROS burden, and limit the propagation of pro-apoptotic signals [27,28,43]. However, excessive or sustained activation of mitophagy may also lead to excessive mitochondrial loss and impair cellular energy reserves and stress adaptability [27,28,41,42,43]. Therefore, future studies should simultaneously evaluate the extent of mitochondrial damage, PINK1/Parkin pathway activity, lysosomal degradation capacity, mitochondrial biogenesis, and energy metabolic status in different disease models to clarify the therapeutic window and causal mechanisms by which H_2_ regulates mitophagy [28,41,42,43].

## 4. Metabolic Reprogramming: Systematic Remodeling of Biochemical Pathways

### 4.1. Glucose Metabolic Reprogramming: Reversal and Bidirectional Regulation of the Warburg Effect

The onset and progression of many diseases are accompanied by reprogramming of cellular energy metabolism, among which glucose-centered metabolic remodeling is regarded as a key driver of pathological change [44,45]. Studies have shown that the regulatory effects of hydrogen on glucose metabolism are context-dependent rather than simply globally suppressive or activating [46,47]. Under abnormal conditions such as oxidative stress or inflammation, hydrogen can help tissues restore metabolic homeostasis by modulating pathways including glycolysis and OXPHOS [45,48]. For example, in a mouse model of SAH, inhalation of 2% hydrogen significantly downregulated HIF-1α expression and inhibited the HIF-1α/MCT4/LDHA axis, thereby reducing lactate accumulation and improving neurological deficits [49]. Activation of HIF-1α could reverse this protective effect of hydrogen, indicating that hydrogen regulates lactate metabolism and energy balance through an HIF-1α-dependent mechanism [50,51,52]. In general, suppression of HIF-1α gene expression by hydrogen may interrupt the aerobic glycolytic pathway required by tumor cells, driving them away from the “Warburg metabolic” state toward a less efficient metabolic mode, ultimately inhibiting cell proliferation and inducing apoptosis [53,54]. However, some studies have reported that in certain ischemia–reperfusion models, such as neonatal piglet brain injury, hydrogen inhalation failed to significantly reduce blood–brain barrier permeability, suggesting that the protective effects of hydrogen may depend on the specific model and administration conditions [55].

In acute ischemia–reperfusion injury such as ischemic heart disease or stroke, hydrogen does not exert its effects by directly modulating glycolysis; rather, it alleviates redox imbalance and protects mitochondrial function, thereby preserving the cellular energy network [45,48]. Specifically, during the ischemic and hypoxic phase, cells rely on glycolysis to meet ATP demands, whereas the massive production of ROS during reperfusion further aggravates energy failure [45,55]. Studies have shown that hydrogen can improve ΔΨm, inhibit ROS generation, and activate the Keap1–Nrf2 antioxidant pathway, thereby attenuating the inhibitory effects of oxidative stress on metabolic enzymes and mitochondrial OXPHOS, helping cells maintain basal energy supply and reducing ischemia–reperfusion injury [45,48]. Taken together, because solid tumors and acutely ischemic tissues differ markedly in oxygen supply and redox status, hydrogen exerts distinct regulatory effects on glucose metabolism under these two pathological conditions: in the tumor microenvironment, sustained stabilization of HIF-1α promotes malignant adaptation toward glycolysis, whereas in acute ischemic states, timely activation of HIF-1α together with the Keap1–Nrf2 pathway helps enhance antioxidant capacity and preserve mitochondrial function, thereby maintaining energy homeostasis and preventing necrosis or apoptosis [48,55].

### 4.2. Lipid Metabolism: Activation of β-Oxidation Driven by PPARα

In addition to dysregulated glucose metabolism, impaired lipid metabolic capacity is also a core pathological basis of metabolic syndrome and non-alcoholic fatty liver disease (NAFLD)/non-alcoholic steatohepatitis (NASH), and is closely associated with defective hepatic fatty acid oxidation and lipid droplet accumulation [56]. A large body of evidence indicates that PPARα is a key transcription factor in maintaining hepatic and systemic energy homeostasis, playing an essential role in the regulation of hepatic fatty acid uptake as well as mitochondrial and peroxisomal β-oxidation [56]. Studies in multiple models of metabolic disease have shown that hydrogen can improve the blood lipid profile and attenuate hepatic steatosis, with activation of the PPARα signaling axis representing a central underlying mechanism [57,58]. Specifically, hydrogen may activate the PGC-1α/PPARα signaling axis, upregulate the expression of fatty acid oxidation–related genes including CPT1A, promote the transport of fatty acids into mitochondria, and enhance β-oxidation, thereby partially restoring impaired lipid metabolic flexibility [36,59]. Because CPT1A is the rate-limiting enzyme for the entry of long-chain fatty acids into mitochondria for β-oxidation, increased expression or activity of CPT1A may help reduce abnormal triglyceride accumulation in the liver and ectopic tissues [60,61]. Animal studies have shown that long-term intervention with HRW can alleviate fatty liver and improve glucolipid metabolic parameters, while mechanistic studies further suggest that PPARα is an important molecular node through which hydrogen regulates lipid metabolism [62,63]. On the other hand, short-term clinical studies have shown that although HRW may reduce hepatic fat deposition or exhibit a trend toward improvement in blood lipid parameters, most lipid indices have not reached statistical significance; therefore, the metabolic benefits of hydrogen in NAFLD may depend on intervention duration, dosage, and the individual’s baseline metabolic status [64,65]. Overall, the remodeling effect of hydrogen on hepatic lipid metabolism appears to rely primarily on the PPARα-centered regulatory axis rather than on nonspecific physical properties such as high lipid solubility [57,58].

**Figure 2:**
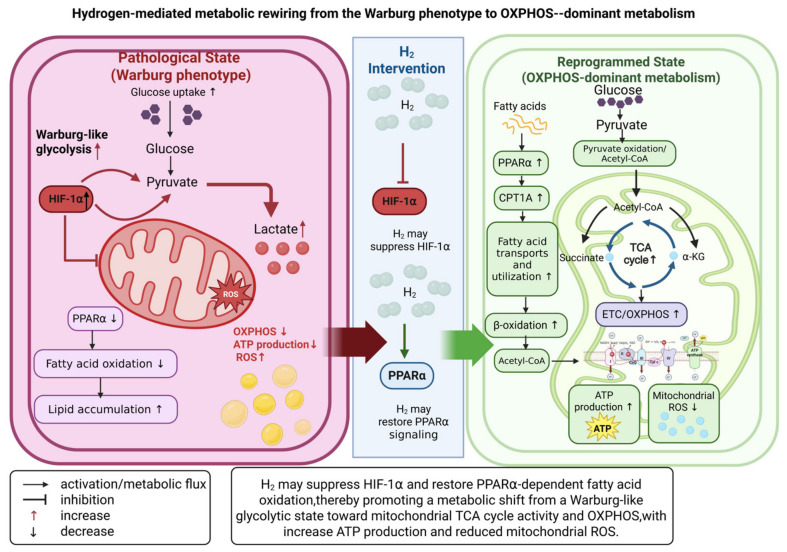
Proposed model of H_2_-mediated metabolic rewiring from a Warburg-like pathological state toward OXPHOS-dominant metabolism.

As shown in Figure 2, in a Warburg-like pathological state, HIF-1α activation favors glycolytic metabolism, increased lactate production, mitochondrial ROS accumulation, and reduced OXPHOS-dependent ATP generation. Meanwhile, reduced PPARα signaling may impair fatty acid oxidation and promote lipid accumulation. H_2_ may partially reverse this metabolic imbalance by suppressing HIF-1α-related glycolytic remodeling and restoring PPARα-dependent fatty acid oxidation. Consequently, pyruvate oxidation, β-oxidation, acetyl-CoA generation, TCA cycle activity, and ETC/OXPHOS function may be enhanced, supporting a shift toward OXPHOS-dominant metabolism.

Under pathological conditions, enhanced HIF-1α activity promotes Warburg-like glycolysis, characterized by increased glucose uptake, pyruvate-to-lactate conversion, lactate accumulation, impaired OXPHOS, reduced ATP production, and increased mitochondrial ROS. Suppression of PPARα is associated with impaired fatty acid oxidation and lipid accumulation. H_2_ intervention may suppress HIF-1α signaling and restore PPARα-dependent fatty acid oxidation. This metabolic shift may enhance fatty acid transport, β-oxidation, acetyl-CoA production, TCA cycle activity, and ETC/OXPHOS function, leading to increased ATP production and reduced mitochondrial ROS. Arrows indicate activation or metabolic flux, blunt-ended lines indicate inhibition, ↑ indicates increase, and ↓ indicates decrease.

## 5. Upstream Regulatory Hub: Integration of the AMPK/Deacetylase Signaling Axis

### 5.1. AMPK as an Essential Molecular Transducer

The MQC and lipid metabolic reprogramming discussed above appear to be sensitive to AMPK-targeted interventions in multiple experimental models. It should be noted, however, that although the commonly used small-molecule inhibitor dorsomorphin, also known as Compound C, can suppress AMPK activity, its structural features and pharmacological profile indicate that it may also affect other signaling pathways, including BMP receptor-related pathways, and exert AMPK-independent effects. Therefore, pharmacological findings obtained using Compound C should be interpreted with caution and further validated by genetic approaches [66,67,68]. In different cell types, inhibition of AMPK activity, for example by Compound C, has been reported to reduce PGC-1α expression and transcriptional activity, as well as attenuate the induction of mitochondrial gene programs, suggesting an important role of AMPK in PGC-1α-mediated transcriptional responses [69,70].

Mechanistically, AMPK acts as a key sensor of cellular energy and nutrient status. Once activated, AMPK can inhibit anabolic processes, stimulate catabolic pathways, and cooperate with the SIRT1–PGC-1α energy-sensing network to promote transcriptional programs involved in mitochondrial biogenesis and oxidative metabolism, while also enhancing cellular antioxidant defense capacity [71,72,73,74]. In addition, AMPK can directly phosphorylate substrates such as ULK1 and mitochondrial fission factor MFF, thereby linking energy stress to the regulation of autophagy, mitophagy, and mitochondrial dynamics. Through these mechanisms, AMPK may coordinate the clearance of damaged mitochondria with the generation of new mitochondria, thereby contributing to the maintenance of MQC [75,76].

In the context of H_2_-related studies, several experimental models have shown that hydrogen treatment is associated with activation of LKB1–AMPK signaling and induction of downstream antioxidant transcriptional responses. Other studies have suggested that H_2_ may upregulate PGC-1α and transcriptional programs related to fatty acid metabolism, while also enhancing PINK1/Parkin-associated mitophagy in stress models such as ischemia–reperfusion injury [58,77,78]. Therefore, a more rigorous interpretation is that current pharmacological and genetic evidence supports the AMPK–PGC-1α axis as a central regulatory node involved in hydrogen-associated MQC and lipid metabolic remodeling. However, whether AMPK activation represents an upstream initiating event in this process remains unclear and requires further clarification through temporal sequence analyses, genetic rescue experiments, and more specific AMPK intervention strategies [42,43,51,67,68,76].

Existing studies further suggest that AMPK may serve as an important signaling mediator in hydrogen-associated metabolic regulation and cytoprotection, rather than merely acting as a secondary downstream effector [79,80]. In both cellular and animal models, hydrogen treatment has been associated with increased AMPK phosphorylation and regulation of downstream biological events, including mTOR signaling, autophagy, antioxidant transcription, and cell survival [55,60]. However, the direct molecular mechanism by which hydrogen modulates AMPK activity has not been fully elucidated. Based on current understanding of AMPK activation, hydrogen may indirectly promote AMPK phosphorylation by affecting mitochondrial function, redox homeostasis, and cellular energy status, thereby altering AMP/ATP and ADP/ATP ratios, enhancing Thr172 phosphorylation of the AMPK α subunit by upstream kinases such as LKB1 and CaMKKβ, and/or inhibiting AMPK dephosphorylation [77,81]. Notably, hydrogen-rich medium has been reported to activate the LKB1–AMPK–FoxO1 signaling pathway without marked ATP depletion, suggesting that hydrogen-induced AMPK activation may not fully depend on the classical energy-deficit mechanism [77]. Moreover, because AMPK can also be regulated through nonclassical mechanisms involving glucose availability, lipid metabolism, lysosomal signaling, and DNA damage responses, the relationship between hydrogen and AMPK is more likely to reflect multilayered network regulation rather than a single linear pathway [81]. As a central intracellular energy sensor, activated AMPK can coordinate metabolic reprogramming, mitochondrial homeostasis, and autophagy, thereby contributing to the cytoprotective effects associated with hydrogen treatment [79,81].

AMPK activation is primarily characterized by phosphorylation at Thr172, and one of its major downstream functions is the regulation of mitochondrial biogenesis and energy metabolic remodeling. On the one hand, activated AMPK can form a functionally coupled axis with SIRT1 and promote the deacetylation and activation of PGC-1α, thereby upregulating factors closely associated with mitochondrial biogenesis, including nuclear respiratory factors NRF-1 and NRF-2, as well as TFAM. These factors contribute to the initiation of mitochondrial transcription and replication programs [82]. On the other hand, AMPK may also influence the epigenetic regulation of PGC-1α and related mitochondrial biogenesis programs by modulating promoter methylation and chromatin accessibility, thereby enhancing the transcriptional activity of PGC-1α and its downstream genes [83]. These changes are often accompanied by increased mtDNA copy number, upregulation of OXPHOS complexes, and improved mitochondrial respiratory function, which is generally consistent with the findings described in the preceding section regarding increased mtDNA copy number and restoration of respiratory chain function [84].

However, the regulatory effects of hydrogen on the AMPK–PGC-1α axis are not entirely consistent across studies. Some evidence suggests that hydrogen can promote autophagy and improve mitochondrial homeostasis through pathways such as AMPK/mTOR signaling [79], whereas other studies have shown that hydrogen can activate LKB1–AMPK signaling without marked ATP depletion, indicating that its mechanism of action may not fully depend on the classical pathway involving an increased AMP/ATP ratio [77]. In light of recent advances in the understanding of classical and nonclassical modes of AMPK activation, it remains unclear whether hydrogen acts directly on upstream AMPK-sensing modules or activates AMPK indirectly by modulating redox status, mitochondrial function, or membrane-related biophysical properties. In addition, differences in hydrogen delivery route, dose, exposure duration, and cell type may also contribute to discrepancies among studies, although this possibility requires further systematic investigation.

In parallel, activated AMPK can participate in lipid metabolic remodeling by regulating the ACC/CPT1A and SREBP-1c pathways. AMPK-mediated phosphorylation of acetyl-CoA carboxylase can reduce malonyl-CoA levels, thereby relieving the inhibitory effect of malonyl-CoA on CPT1A and promoting fatty acid β-oxidation [85]. At the same time, AMPK-mediated phosphorylation and inhibition of SREBP-1c can decrease the expression of genes involved in de novo lipogenesis, including FASN, SCD1, and molecules associated with triglyceride synthesis [86]. These mechanisms are consistent with the broader role of AMPK in limiting lipid accumulation and restoring lipid metabolic homeostasis [85,86]. In addition, lipid droplet dynamics are closely associated with cellular lipid metabolic status and may reflect the balance between lipid storage, mobilization, and oxidative utilization [87]. Nevertheless, AMPK-dependent lipid metabolic phenotypes vary across models, disease stages, and intervention conditions [88]. Therefore, the regulation of AMPK-associated lipid metabolic remodeling by hydrogen is likely to be context-dependent, and its precise molecular mechanisms remain to be further clarified.

### 5.2. Deacetylation Networks and the Rapid Metabolic Responses of Sirtuins

Existing evidence suggests that AMPK is more likely to act as one of the key central nodes in hydrogen-induced metabolic remodeling rather than as a peripheral factor in metabolic regulation. However, current evidence remains insufficient to directly define AMPK as the “common upstream pathway” or “core transducer” of hydrogen-mediated metabolic regulation. A more appropriate interpretation is that hydrogen may indirectly promote AMPK activation by influencing cellular redox status, mitochondrial function, and energy stress. Nevertheless, whether hydrogen necessarily induces AMPK Thr172 phosphorylation through changes in membrane lipid properties, depolarization of ΔΨm, and an increased AMP/ATP ratio has not been consistently confirmed across different experimental models [31,71,89,90,91]. As a classical energy sensor, AMPK plays an important role in maintaining metabolic homeostasis, promoting adaptive mitochondrial function, and regulating autophagy. Therefore, it is more appropriate to describe AMPK as a high-probability integrative node in the biological effects of hydrogen, rather than as the only fully established initiating switch [71,91].

After AMPK activation, its downstream signaling may proceed mainly through two closely related directions: mitochondrial biogenesis and transcriptional reprogramming. On the one hand, AMPK can promote the deacetylation of PGC-1α and FOXO family transcription factors by increasing NAD^+^ availability and enhancing SIRT1 activity, thereby upregulating the expression of genes related to mitochondrial biogenesis, including NRF-1, NRF-2, and TFAM. On the other hand, some studies suggest that AMPK may also attenuate the inhibitory effects of certain epigenetic regulators on promoters associated with mitochondrial metabolism through direct phosphorylation or indirect regulation, thereby facilitating the establishment of adaptive metabolic transcriptional programs. However, direct evidence at this level still relies heavily on specific experimental models and remains insufficient to support its generalization as a universal mechanism [72,92]. In other words, AMPK is an important signaling hub linking changes in cellular energy status to mitochondrial remodeling, rather than the sole direct upstream regulator of all epigenetic events [72,91,92].

In addition, AMPK can suppress acetyl-CoA carboxylase (ACC) through phosphorylation, thereby reducing intracellular malonyl-CoA levels, relieving its inhibitory effect on CPT1A, and promoting fatty acid β-oxidation [86,93]. At the same time, AMPK can inhibit the maturation and activation of SREBP-1c and reduce the expression of its downstream lipogenic genes, including FASN, SCD1, and DGAT2, thereby shifting cellular metabolism from an anabolic state toward a more oxidative metabolic state [86]. This mechanistic framework is generally consistent with phenomena such as reduced lipid droplet accumulation and lipid metabolic remodeling. However, the magnitude, persistence, and degree of AMPK dependence of these effects may vary across different disease models, pathological stages, and intervention conditions. Therefore, AMPK-associated lipid metabolic remodeling should be regarded as an important candidate mechanism contributing to the metabolic effects of hydrogen, rather than as a universally established mechanism under all conditions [86,91,93].

In addition to phosphorylation cascades, hydrogen-mediated metabolic remodeling may also be associated with the NAD^+^/Sirtuins axis. Existing studies have shown that AMPK can enhance SIRT1/SIRT3 activity by promoting NAD^+^ regeneration and, together with target molecules such as PGC-1α and FOXO, form a cooperative network that regulates energy metabolism and redox homeostasis [72,92,94]. In this AMPK–NAD^+^–Sirtuins network, AMPK activation increases NAD^+^ availability, thereby enhancing Sirtuins activity, which in turn further improves mitochondrial function and metabolic adaptability [72,92]. However, some studies have also indicated that SIRT3 is not an absolutely essential requirement for the protective effects of related interventions under all conditions. Therefore, this positive feedback loop should be regarded as a relatively common, but not universally applicable, mechanistic module [94,95,96].

In various models of oxidative stress and metabolic dysregulation, the deacetylase activity of SIRT3 is not confined to a single metabolic step. Instead, SIRT3 plays an important integrative regulatory role between mitochondrial energy metabolism and antioxidant defense. Existing studies have shown that SIRT3 can coordinate the regulation of the tricarboxylic acid (TCA) cycle, OXPHOS, NADPH maintenance, and ROS clearance by deacetylating multiple mitochondrial metabolic enzymes and antioxidant-related proteins, thereby helping to preserve mitochondrial homeostasis under pathological stress conditions [97,98]. However, current evidence suggests that SIRT3 is more likely to function as an important regulatory node in these processes, rather than as an absolute determinant that ensures the synchronized operation of energy metabolism and antioxidant defense in all models. The magnitude of its specific effects may still be influenced by factors such as tissue type, degree of injury, and NAD^+^ metabolic status [95,98,99]. In addition, compared with transcriptional regulation, post-translational modifications such as protein deacetylation generally exhibit faster response kinetics and may therefore represent one of the candidate mechanisms explaining the early improvement in mitochondrial redox status after hydrogen intervention. Based on the current evidence, the AMPK–Sirtuins–mitochondrial enzyme pathway may constitute an important mechanistic module involved in the early phase of the metabolic effects of hydrogen. However, whether it represents a unified molecular basis for the rapid onset of hydrogen action still requires further validation through more temporally resolved and causally oriented studies [91,94,95,98,99].

**Figure 3:**
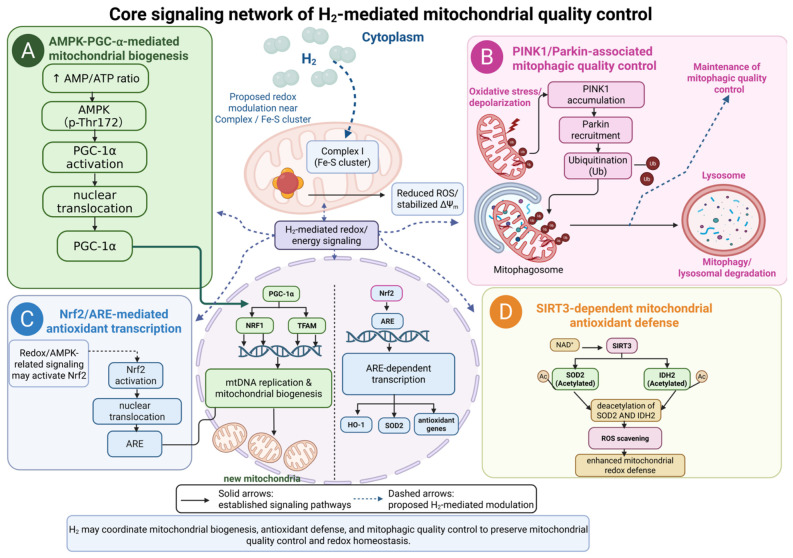
Core signaling network underlying H_2_-mediated mitochondrial quality control. H_2_ may coordinate MQC through multiple interconnected pathways involving mitochondrial biogenesis, mitophagy, antioxidant transcription, and mitochondrial redox defense. (**A**) AMPK–PGC-1α-mediated mitochondrial biogenesis: increased AMP/ATP ratio activates AMPK, which promotes PGC-1α activation and nuclear translocation, thereby regulating NRF1/TFAM-dependent mtDNA replication and mitochondrial biogenesis. (**B**) PINK1/Parkin-associated mitophagic quality control: oxidative stress and mitochondrial depolarization promote PINK1 accumulation, Parkin recruitment, ubiquitination, mitophagosome formation, and lysosomal degradation of damaged mitochondria. (**C**) Nrf2/ARE-mediated antioxidant transcription: redox- or AMPK-related signaling may activate Nrf2, promote its nuclear translocation, and induce ARE-dependent antioxidant genes such as HO-1 and SOD2. (**D**) SIRT3-dependent mitochondrial antioxidant defense: NAD^+^-dependent SIRT3 activation promotes deacetylation of mitochondrial antioxidant enzymes, including SOD2 and IDH2, thereby enhancing ROS scavenging and mitochondrial redox defense. Solid arrows indicate established signaling pathways, whereas dashed arrows indicate proposed H_2_-mediated modulation.

As shown in Figure 3 H_2_-mediated MQC may involve four coordinated signaling modules. First, H_2_ may activate AMPK–PGC-1α-related mitochondrial biogenesis, thereby promoting NRF1/TFAM-dependent mtDNA replication and the generation of new mitochondria. Second, H_2_ may support PINK1/Parkin-associated mitophagic quality control, facilitating the removal of damaged mitochondria through mitophagosome formation and lysosomal degradation. Third, H_2_ may enhance Nrf2/ARE-mediated antioxidant transcription, leading to increased expression of antioxidant genes such as HO-1 and SOD2. Fourth, H_2_ may strengthen SIRT3-dependent mitochondrial antioxidant defense by promoting the deacetylation of SOD2 and IDH2, thereby enhancing ROS scavenging and mitochondrial redox homeostasis.

## 6. Validation by Preclinical and Clinical Evidence

### 6.1. Metabolic Diseases: Bioenergetic Restoration and Quantification of Insulin Sensitivity

In translational studies targeting NAFLD and T2DM, the efficacy evaluation system is gradually shifting from a single histopathological endpoint toward multidimensional functional endpoints. In addition to conventional indicators such as histological scores, blood glucose, and blood lipid levels, researchers are increasingly incorporating mitochondrial respiration, ATP production, OXPHOS efficiency, and insulin sensitivity–related parameters into integrated evaluation systems to more comprehensively reflect the metabolic responses following hydrogen intervention [100,101]. In NAFLD and T2DM, mitochondrial bioenergetic abnormalities are generally closely associated with lipid accumulation, enhanced oxidative stress, and insulin resistance, and are mainly manifested by disordered ETC function, reduced ATP synthesis efficiency, and impaired redox homeostasis [101,102]. However, it should be recognized that mitochondrial oxidative function does not necessarily decline continuously across different models and disease stages. Some studies suggest that oxidative metabolism may show a transient increase during the early or compensatory stages of disease. Therefore, mitochondrial dysfunction can be regarded as a key mechanistic module in the progression of metabolic diseases, but not as a single decisive event that changes in a uniform direction under all conditions [102,103].

For NAFLD and metabolic syndrome induced by a high-fat diet, existing animal experiments and early clinical studies suggest that hydrogen intervention may improve mitochondrial bioenergetic status, with its effects being more consistent with mitochondrial functional reorganization rather than a fully established systemic remodeling process. In experimental models, hydrogen treatment can enhance OXPHOS coupling efficiency and ATP-generating capacity, accompanied by partial recovery of mitochondrial respiratory parameters, increased CoQ levels, and improved redox status. Some studies further suggest that this process may involve increased Complex I/II-related respiratory flux and optimization of cellular redox balance, thereby alleviating the bioenergetic imbalance caused by lipid overload [29,102,104]. However, there is still insufficient and inconsistent direct evidence regarding whether the NAD^+^/NADH ratio undergoes stable changes, whether the proteomic profile related to energy metabolism is systematically corrected, and whether these alterations are consistent across different models. Therefore, these changes should be regarded as potentially involved mechanistic aspects, rather than being defined as fully confirmed common pathways [29,103,104]. At the clinical level, after more than 8 weeks of HRW intervention, patients with NAFLD showed improvements in platelet mitochondrial bioenergetic function and CoQ10 levels; in addition, a 13-week randomized controlled study of hydrogen–oxygen inhalation demonstrated improvements in liver fat content, blood lipid profiles, and liver enzyme indices [100,105]. However, it should be noted that most mitochondrial-related readouts in current human studies are derived from peripheral surrogate markers or indirect endpoints, and therefore cannot be considered fully equivalent to the direct restoration of mitochondrial OXPHOS function within hepatocytes. Overall, hydrogen may exert metabolic protective effects by improving the redox environment and certain mitochondrial bioenergetic parameters, and its “mitochondria-targeted” characteristics have received some degree of clinical support, although the causal chain linking preclinical mechanisms to human outcomes still requires further refinement [100,103,105].

At the same time, serum β-hydroxybutyrate (β-HB), as one of the important indicators of ketone body metabolism, is often used as a surrogate readout for assessing hepatic fatty acid oxidation and ketogenesis activity. Under healthy conditions, its changes generally correspond well with the level of hepatic fatty acid β-oxidation; however, in the context of obesity and NAFLD, although this relationship still retains some reference value, it may also be influenced by factors such as nutritional status, disease stage of the liver, and mitochondrial metabolic adaptation. Therefore, β-HB is better regarded as an indicative marker reflecting metabolic changes, rather than being simply considered an absolute equivalent readout of mitochondrial fatty acid oxidation flux [106]. In models of high-fat diet and related metabolic disorders, elevated β-HB is often accompanied by enhanced lipid oxidation, reduced lipid droplet burden, and improvement in certain mitochondrial respiratory parameters, suggesting that fuel utilization may shift from a pattern dominated by lipid storage toward a metabolic state more oriented toward fatty acid mobilization and ketone production. However, this change should be understood more appropriately as an adaptive metabolic redistribution, rather than being taken as evidence of “fundamental reprogramming” solely on the basis of elevated β-HB [103,106]. With regard to hydrogen intervention, existing animal experiments and some clinical studies have shown that it can improve lipid deposition, redox status, and mitochondria-related metabolic parameters [22,104,105]. However, direct evidence using β-HB or ketogenesis as the primary endpoint to demonstrate that hydrogen can stably activate the “fatty acid oxidation–ketogenesis” pathway remains limited. Therefore, at the present stage, it may be considered that hydrogen could indirectly drive the body toward a metabolic state more favorable for energy output by improving the redox environment and promoting fatty acid oxidation–related metabolic adaptation, but whether this process necessarily takes ketogenesis as its core feature still requires more direct evidence [23,104,105].

In addition, metabolic improvements at the levels of the liver, adipose tissue, and skeletal muscle may collectively contribute to the restoration of systemic insulin sensitivity. However, functional improvement at the tissue level cannot be simply regarded as equivalent to a synchronous decline in clinical indicators of insulin resistance [23,107,108]. Existing randomized controlled trials and real-world studies have shown that medium- to long-term consumption of HRW or inhalation of hydrogen can improve certain glucolipid metabolic parameters, and a reduction in HOMA-IR has been observed in some cohorts [23,108]. However, some randomized controlled studies have not found a significant between-group difference in HOMA-IR improvement [107]. Overall, hydrogen has shown some potential in improving metabolic risk factors and certain parameters related to insulin resistance, but the magnitude of its effect, the populations to which it applies, and the reproducibility of the findings still require further clarification through higher-quality studies [23,107,108].

Current clinical studies suggest that hydrogen intervention may show potential value in improving systemic glucose and lipid homeostasis by regulating the redox status, energy metabolism, and inflammatory responses of multiple metabolically relevant tissues and organs, thereby providing preliminary evidence to support its clinical translation [23,105,107,108,109]. However, it should also be recognized that existing studies show substantial heterogeneity in population composition, route of administration, intervention dose, follow-up duration, and endpoint design, and that most studies have relatively small sample sizes, with some conclusions derived mainly from exploratory trials, retrospective studies, or real-world data. Therefore, the current evidence base remains insufficient to support clear clinical recommendations or practical guidelines. In the future, high-quality randomized controlled trials with larger sample sizes, longer follow-up periods, and more rigorous study designs are still needed to further clarify the long-term efficacy, safety, target populations, and clinical application boundaries of hydrogen intervention.

### 6.2. Neurodegenerative Diseases: Mitochondrial Complex Activity and Cerebral Energy Reserve

The reported metabolic and bioenergetic effects of H_2_ across different disease models are summarized in Table 2. Overall, H_2_ appears to influence several common mitochondrial and metabolic endpoints, including OXPHOS, mitochondrial membrane potential, ATP production, redox homeostasis, fatty acid oxidation, and glycolytic remodeling. In metabolic and inflammatory disease models, H_2_ is mainly associated with improved mitochondrial function, reduced oxidative stress, and restoration of systemic metabolic homeostasis. In neurological injury, ischemia–reperfusion injury, and tumor-related settings, the effects of H_2_ appear more context-dependent and may involve both mitochondrial protection and disease-specific metabolic remodeling [3,9,10,22,29,31,35,45,104,105,106,107,108,109,110,111,112,113,114,115,116,117,118,119,120,121,122].

Based on these disease-specific findings, the following sections further discuss the potential roles of H_2_ in neurodegenerative diseases, ischemia–hypoxia-related injury, and tumor metabolic regulation.

For neurodegenerative diseases such as Parkinson’s disease (PD) and Alzheimer’s disease (AD), existing studies suggest that the neuroprotective effects of hydrogen may depend in part on the maintenance of mitochondrial function, improvement of redox homeostasis, and adaptation of energy metabolism, rather than being limited solely to the regulation of a single step in the ETC [3,31,109,110]. In vitro studies have shown that the effects of hydrogen on mitochondrial electron flow exhibit a certain degree of state dependence and may be modulated by the substrate environment and the NAD^+^/NADH ratio. Under specific experimental conditions, hydrogen can suppress approximately 50% of superoxide anion production derived from Complex I, accompanied by a mild decrease in ΔΨm, suggesting that it may alleviate oxidative damage by reducing abnormal electron leakage [95]. However, these findings are derived mainly from ex vivo or cellular experiments and therefore cannot yet be directly regarded as a universal mechanism in neurodegenerative diseases. Furthermore, in multiple models of nervous system injury and neurodegenerative disease, hydrogen intervention is frequently accompanied by reduced ROS levels, improved mitochondrial function, and attenuated apoptosis, indicating that it may ameliorate neuronal bioenergetic crisis by simultaneously relieving ETC dysfunction and oxidative stress [3,110,111]. Overall, hydrogen may participate in the neuroprotective process in neurodegenerative diseases by enhancing mitochondrial electron transport efficiency, reducing abnormal electron leakage, and alleviating secondary oxidative damage, but its direct regulatory effects on specific ETC complexes, as well as its functional characteristics at different stages of PD and AD progression, still require further investigation [3,109,110].

Under conditions of ischemia–hypoxia or neurotoxic stress, hydrogen-related interventions may exert neuroprotective effects by preserving mitochondrial function and improving cerebral energy metabolism [45,110,112,123]. In models of cerebral ischemia–reperfusion and sepsis-associated brain injury, hydrogen can partially restore ΔΨm, increase ATP levels, and attenuate oxidative damage, and these changes are consistent with reduced neuronal injury and improved cognitive function [112,113,123]. At the same time, metabolomic and mass spectrometry imaging studies after cerebral ischemia have shown that disruption of the TCA cycle and spatially heterogeneous alterations in the ATP/ADP/AMP energy pool are important features of cerebral energy crisis [114,115]. Therefore, hydrogen may help maintain cerebral energy supply by protecting mitochondria and alleviating secondary disturbances in energy metabolism, although its direct regulatory effects on specific metabolic pathways remain to be further clarified [3,45,110,111].

In the field of tumor metabolic intervention, no consensus has yet been reached regarding the effects of hydrogen on the Warburg phenotype. Relevant studies suggest that hydrogen may indirectly influence glycolysis-related metabolic processes by modulating redox status, mitochondrial function, and the tumor microenvironment; however, whether it can stably reduce lactate accumulation, alleviate microenvironmental acidification, and broadly weaken tumor cell dependence on aerobic glycolysis still requires more direct investigation [9,116,117,118]. Given the important roles of lactate accumulation and an acidic microenvironment in tumor progression and immune suppression, the metabolic effects associated with hydrogen merit further study, but current evidence remains insufficient to regard it as a confirmed strategy for reversing the Warburg effect [122,123]. At the same time, some studies have also observed that hydrogen can promote the proliferation of certain specific cancer cells, indicating that its effects may show marked dependence on tumor type and metabolic background [10,119].

At the clinical level, ^18^F-FDG PET/CT can reflect tumor glucose metabolic activity through parameters such as SUVmax, MTV, and TLG, and has been widely applied in treatment response monitoring and prognostic assessment [120,121]. However, it should be noted that changes in SUVmax are, in essence, still indirect imaging readouts and cannot be simply equated with true changes in glycolytic flux or cellular glucose utilization [120]. With regard to hydrogen-related interventions, current clinical studies still mainly use endpoints such as CT/MRI findings, symptomatic improvement, and changes in tumor markers, whereas studies directly demonstrating that hydrogen can reduce tumor glucose metabolic burden on the basis of ^18^F-FDG PET/CT remain relatively scarce [121,122]. Therefore, the clinical imaging effects of hydrogen on tumor metabolism still need to be further clarified through more rigorously designed prospective studies.

Taken together, the evidence summarized in Table 2 suggests that H_2_-related metabolic regulation is highly disease- and context-dependent. Although common mitochondrial endpoints such as ΔΨm, ATP production, ROS levels, and respiratory chain function are frequently involved, the direction and magnitude of these effects vary across disease models, administration routes, and experimental conditions [3,9,10,22,29,31,35,45,104,105,106,107,108,109,110,111,112,113,114,115,116,117,118,119,120,121,122].

## 7. Conclusions and Future Perspectives

### 7.1. Core Conclusion: Metabolic Reprogramming Is a Fundamental Mechanism Underlying the Therapeutic Effects of Hydrogen

Re-examining the biological properties of hydrogen reveals that characterizing it merely as an “antioxidant” is no longer sufficient to comprehensively capture its functional features. Current studies generally suggest that the biological effects of hydrogen are closely associated with the coordinated regulation of redox homeostasis, mitochondrial function, and energy metabolic networks, with mitochondria likely serving as one of its key functional hubs [9,112]. In multiple disease models, metabolic pathways and regulators such as AMPK, Sirtuins, PGC-1α, PPARα, and fatty acid oxidation/OXPHOS have been repeatedly observed to participate in hydrogen-related effects, suggesting that hydrogen may improve cellular bioenergetic status and, to some extent, correct abnormalities in glucose and lipid metabolism by modulating the continuous process of “energy sensing–MQC–metabolic flux remodeling” [9,112,118]. However, no consensus has yet been reached regarding the upstream sensing mechanisms of hydrogen, its direct mode of action on ΔΨm or redox status, or the specificity of its signal [124] transduction across different tissues and disease contexts [9,112,118]. Particularly in the field of cancer, the effects of hydrogen on the Warburg phenotype are not entirely consistent, and some studies have even suggested that it may promote cancer cell proliferation under specific metabolic conditions, indicating a marked model dependency of its actions [10,116,118]. Overall, metabolic reprogramming is likely one of the important common mechanisms through which hydrogen exerts its broad biological effects, but it cannot yet be regarded as the sole fundamental mechanism that has been fully established. Future studies integrating metabolic flux analysis, spatiotemporally resolved assessment of mitochondrial function, and prospective clinical research are still needed to further clarify its causal chain and boundaries of applicability [9,112,121].

### 7.2. Current Challenges and Future Perspectives

Although the metabolic effects associated with hydrogen have been extensively described at multiple levels, including AMPK, mitochondrial function, redox homeostasis, and downstream transcriptional regulation, these lines of evidence more often reflect biological outcomes after signal transduction rather than the key events occurring at the initiation stage of action. Therefore, the direct molecular targets of hydrogen remain unidentified to date, which constitutes one of the most critical mechanistic gaps in the field [2,3]. Because H_2_ exhibits relatively limited chemical reactivity under physiological conditions, traditional physicochemical models remain insufficient to fully explain how it can trigger broad yet relatively specific signal transduction at extremely low concentrations. To address this issue, some scholars have proposed more refined biophysical hypotheses in recent years. On the one hand, hydrogen may affect metal cofactors or electron transfer nodes within the mitochondrial ETC that are highly sensitive to redox status, such as Fe-S clusters or heme-related sites. On the other hand, hydrogen may also indirectly regulate protein conformational dynamics and allosteric coupling of membrane proteins by altering the structural properties of interfacial water or hydration layers surrounding biomacromolecules. However, these mechanisms currently remain largely at the level of theoretical inference or narrative discussion in review articles, and still lack direct evidence from structural biology and in situ functional studies [2,3]. Future research should place greater emphasis on identifying the initial physicochemical events through which hydrogen exerts its effects, rather than merely continuing to expand downstream pathway descriptions, as this will help advance hydrogen medicine research from phenomenological observation toward a more rigorous mechanistic framework [2,3].

At the methodological level, traditional metabolomics based on tissue homogenates usually provides only overall average readouts and is therefore insufficient to reveal the complex spatial heterogeneity within lesions. In recent years, technologies such as spatial metabolomics, spatiotemporal metabolomics, and in situ mass spectrometry imaging have continued to advance and have shown clear advantages in studies of the tumor microenvironment and ischemic brain tissue. These approaches can depict the spatial distribution of metabolites and their dynamic changes while preserving the structural context of tissues [123,124,125]. This is particularly important for research on the biological effects of hydrogen, because responses to hydrogen are likely to differ across distinct microregions. For example, the hypoxic core and normoxic periphery of tumors, as well as the ischemic penumbra and adjacent relatively normal brain tissue, may differ in metabolic sensitivity, redox thresholds, and the kinetics of bioenergetic remodeling [123,124,125]. Therefore, constructing high-resolution spatiotemporal metabolic maps may provide a more testable mechanistic framework for explaining the “bidirectional regulation” exerted by hydrogen and may also lay the foundation for stratified intervention and individualized translational research. However, this direction still requires further validation through more rigorous in situ detection, dynamic tracking, and clinically correlated studies [123,124,125].

## Figures and Tables

**Table 1:** Pharmacokinetic characteristics, tissue distribution, application scenarios, and limitations of different H_2_ administration routes.

Administration Method	Pharmacokinetic Characteristics	Tissue Distribution/Delivery Advantages	Typical Application Scenarios	Limitations	References
H_2_ inhalation	Rapid pulmonary absorption and rapid systemic exposure; arterial H_2_ concentration rises quickly after inhalation and decreases rapidly after cessation. Continuous inhalation allows relatively controllable exposure duration.	Direct delivery through the alveoli and pulmonary circulation; measurable distribution to multiple organs and tissues, including highly perfused organs such as lung, brain, liver, and kidney.	Acute inflammatory or critical injury models, especially LPS-induced acute lung injury and polymicrobial sepsis; potentially suitable for short-term or emergency intervention requiring rapid systemic exposure.	Requires gas-delivery equipment or an H_2_ generator; dose, concentration, and exposure duration need careful control; safety issues related to H_2_ flammability should be considered.	PK: [15,27,28]; Distribution: [6,15,28]; Applications: [11,14]; Limitations: [27,28]
Oral HRW	Noninvasive oral administration; H_2_ exposure is rapid but transient after ingestion, and depends on dissolved H_2_ concentration, intake volume, preparation method, and storage conditions.	Direct gastrointestinal exposure; absorbed H_2_ can enter the portal circulation and may preferentially expose the gastrointestinal tract and liver. Repeated intake is feasible for longer-term intervention.	Chronic or preventive intervention, especially metabolic disorders such as impaired glucose metabolism, type 2 diabetes, dyslipidemia, and metabolic syndrome; also used in maternal inflammation-related fetal injury models.	Dissolved H_2_ concentration decreases during storage and handling; dose standardization is difficult; systemic exposure is relatively short-lived and may be insufficient for acute high-level systemic delivery.	PK: [27,28,29,30]; Distribution: [28,29,30]; Applications: [12,31,32]; Limitations: [28,29,30]
Hydrogen-rich saline/injection	Parenteral administration bypasses oral intake and allows more controlled dosing in experimental settings; intravenous infusion and intraperitoneal injection show route-dependent H_2_ kinetics.	Allows direct systemic or local delivery depending on the injection route; intraperitoneal administration may provide exposure through abdominal and portal circulation, whereas intravenous infusion provides direct vascular delivery.	Mainly used in preclinical or experimental organ injury models, including endotoxin-induced lung dysfunction, intestinal ischemia–reperfusion injury, renal ischemia–reperfusion injury, and cerebral ischemia models.	Invasive administration; requires sterile preparation and clinical supervision; translational evidence remains limited; H_2_ distribution may be route-dependent and not always sufficient for distant organs.	PK: [27,33,34]; Distribution: [27,33,34]; Applications: [13,32,35,36]; Limitations: [27,28,33,34]

Note: This table summarizes the kinetic differences in the three main routes of administration. Inhalation method through the pulmonary circulation to achieve rapid distribution of the whole body, suitable for acute ischemic disease; drinking HRW has unique ‘pulse’ absorption characteristics and can induce the secretion of ghrelin, which is more suitable for the long-term management of chronic metabolic diseases. The injection method provides precise dose control and is often used in experimental treatment of specific organ damage. Abbreviation: Cmax, peak plasma concentration; ppm, million-percent concentration.

**Table 2:** Key metabolic targets, bioenergetic/metabolic alterations, and clinical/pathological implications of hydrogen intervention across different disease models.

**Disease Model**	**Key Metabolic Target**	**Changes in Bioenergetic/Metabolic Indicators**	**Clinical/Pathological Significance**	**References**
Nonalcoholic fatty liver disease (NAFLD)	Mitochondrial OXPHOS; hepatic lipid metabolism; fatty acid oxidation	ATP/ADP ratio ↑ or normalized; serum β-hydroxybutyrate ↑ in some studies; fatty acid oxidation ↑; hepatic lipid accumulation ↓; steatosis-related injury ↓	Associated with improved hepatic bioenergetic status and attenuation of steatosis	[29,104,105]
Metabolic syndrome/type 2 diabetes-related metabolic dysfunction	Insulin sensitivity; glucose and lipid metabolism; redox homeostasis	HOMA-IR ↓; insulin sensitivity ↑; fasting glucose/HbA1c ↓ in some studies; lipid profile improved; inflammatory/redox biomarkers ↓	Suggests improvement in systemic glucose–lipid metabolic homeostasis	[22,107,108]
Neurodegenerative diseases	Mitochondrial electron transport chain (ETC); complex I-related redox regulation; ΔΨm maintenance	Complex I-associated superoxide generation ↓; ΔΨm loss ↓; ATP reserve ↑ or partially restored; mitochondrial respiratory activity ↑ in some models	Associated with preservation of neuronal mitochondrial function and attenuation of neurotoxic injury	[3,31,110,111]
Sepsis/inflammation-associated injury	Mitochondrial dysfunction; redox homeostasis; inflammatory signaling	ROS ↓; oxidative stress markers ↓; GSH/GSSG ratio ↑ or normalized; mitochondrial dynamics/function improved; inflammatory injury markers ↓	Associated with restoration of antioxidant capacity and attenuation of inflammation-related mitochondrial injury	[35,112]
Ischemia/reperfusion injury	Mitochondrial function; oxidative stress; energy metabolism	Mitochondrial dysfunction ↓; oxidative injury ↓; ATP depletion ↓; ΔΨm loss ↓; metabolic disturbance partially improved	Supports a protective role of H_2_ in ischemia/reperfusion-related mitochondrial injury	[45,113,114,115]
Advanced cancer	Tumor glycolytic phenotype; Warburg-associated metabolism; mitochondrial stress responses	Lactate production ↓ in some settings; FDG uptake/SUV-related parameters ↓ or altered in limited studies; mitochondrial stress response altered; effects are model-dependent	Suggests a potential role in tumor metabolic regulation, but current evidence is insufficient to support stable reversal of the Warburg phenotype	[9,10,116,117,118,119,120,121,122]

Table note: This table summarizes the major metabolic effects of hydrogen intervention across different disease models, including NAFLD, metabolic syndrome, neurodegenerative diseases, advanced cancer, and sepsis/inflammation. The table focuses on key metabolic targets, representative bioenergetic or metabolic changes, and their corresponding clinical/pathological implications. Overall, hydrogen-related effects are mainly associated with mitochondrial OXPHOS, insulin sensitivity, ETC function, Warburg-related metabolism, and redox homeostasis. However, substantial heterogeneity exists among studies with respect to disease background, intervention route, dose, duration, and endpoint definition; therefore, some reported changes should be interpreted in the context of specific models and levels of evidence. ↑ indicates increase, activation, improvement, or restoration; ↓ indicates decrease, inhibition, reduction, or attenuation. The effects summarized in this table are derived from heterogeneous preclinical and clinical studies and should be interpreted in a model- and context-dependent manner.

## Data Availability

No new data were created or analyzed in this study.

## Abbreviations

The following abbreviations are used in this manuscript:

H_2_	Molecular hydrogen
ROS	reactive oxygen species
RNS	reactive nitrogen species
MQC	Mitochondrial Quality Control
AMPK	AMP-Activated Protein Kinase
UPRmt	unfolded protein response
OXPHOS	oxidative phosphorylation
HRW	Hydrogen-Rich Water
PK	Pharmacokinetics
LPS	Lipopolysaccharide
IMM	inner mitochondrial membrane
ETC	electron transport chain
ΔΨm	mitochondrial membrane potential
PCET	proton-coupled electron transfer
SAH	subarachnoid hemorrhage
HO-1	heme oxygenase-1
SOD2	mitochondrial superoxide dismutase 2
PGC-1α	Peroxisome Proliferator-Activated Receptor Gamma Coactivator 1-Alpha
TFAM	mitochondrial transcription factor A
Drp1	dynamin-related protein 1
MFF	mitochondrial fission factor
Mfn1/2	mitofusin 1/2
OPA1	optic atrophy 1
PINK1	PTEN-induced kinase 1
OGD/R	oxygen-glucose deprivation/reoxygenation
CLP	cecal ligation and puncture
TEM	transmission electron microscopy
NAFLD	non-alcoholic fatty liver disease
NASH	non-alcoholic steatohepatitis
CPT1A	Carnitine Palmitoyltransferase 1A
TCA	Tricarboxylic Acid Cycle
ULK1	Unc-51 Like Autophagy Activating Kinase 1
FASN	Fatty Acid Synthase
SCD1	Stearoyl-CoA Desaturase 1
T2DM	Type 2 Diabetes Mellitus
β-HB	β-hydroxybutyrate
PD	Parkinson’s disease
AD	Alzheimer’s disease
